# Time trends of mortality in patients with non-functioning pituitary adenoma: a Swedish nationwide study

**DOI:** 10.1007/s11102-016-0764-8

**Published:** 2016-10-14

**Authors:** Daniel S. Olsson, Ing-Liss Bryngelsson, Oskar Ragnarsson

**Affiliations:** 1 Department of Endocrinology, Institute of Medicine, Sahlgrenska Academy, University of Gothenburg and Sahlgrenska University Hospital, Gröna Stråket 8, 413 45 Gothenburg, Sweden; 2 Department of Occupational and Environmental Medicine, Örebro University Hospital, 701 85 Örebro, Sweden

**Keywords:** Mortality, Non-functioning pituitary adenoma, Women, Time trends, Hypopituitarism, Surgery

## Abstract

**Purpose:**

Patients with non-functioning pituitary adenomas (NFPA), especially women, have increased mortality. The aim of this study was to investigate whether mortality in NFPA patients has changed during the last two decades.

**Methods:**

This was a nationwide population-based study including 2795 patients (1502 men, 1293 women) diagnosed with NFPA between 1997 and 2011. Patients were identified and followed in Swedish National Health Registries. Standardized mortality ratios (SMRs) with 95 % confidence intervals were calculated for three time periods at first NFPA diagnosis using the general population as reference.

**Results:**

Mean (±SD) age at NFPA diagnosis was 58.9 ± 16.8 years. Mean (range) follow-up time was 8.3 (0–18) years, resulting in 20,517 patient-years at risk. Surgical treatment and radiotherapy were used in 53 and 5 %, respectively. The prevalence of hypopituitarism was 64 % during the first time period of diagnosis and then declined gradually during the study period (*P* value for trend <0.0001). The use of pituitary surgery and radiotherapy remained stable. In women, mortality was increased for patients diagnosed between 1997 and 2006 but not for those diagnosed between 2007 and 2011. The SMR in men remained stable throughout the study and did not differ from the general population. During the last time period, 2007–2011, the SMR between men and women did not differ.

**Conclusions:**

While mortality in men with NFPA remains normal and stable during the last two decades, mortality in women has declined. Decreasing prevalence of pituitary insufficiency may be a plausible explanation for this positive development.

## Introduction

Hypopituitary patients have increased mortality [1–4]. Several factors associated with increased risk have been suggested such as etiology, inadequate hormone replacement treatment, pituitary radiotherapy, transcranial surgery, and diagnosis of hypopituitarism at young age [2, 3, 5–7]. Furthermore, women with pituitary insufficiency have frequently been found to have an increased mortality compared to men [1, 3, 8].

We have recently demonstrated that patients, particularly women, with non-functioning pituitary adenoma (NFPA), have an increased mortality compared to the background population [5]. We have also shown that women with NFPA have a higher incidence of type 2 diabetes mellitus, myocardial infarction, cerebral infarction, and fractures in comparison to both the general population and to men [9].

In the course of the past two decades substantial progress has been achieved in terms of diagnosis, treatment, and surveillance of patients with pituitary insufficiency. This includes constantly improving imaging techniques, establishment of growth hormone as a part of the replacement therapy regime, and adjustment of the glucocorticoid replacement therapy towards a near-physiological pattern [10–13]. Moreover, the current surgical approaches for lesions in the pituitary region have also proven more accurate and less traumatic [14].

A meta-analysis, comparing studies including patients diagnosed during different time periods, has shown that patients diagnosed during the 1950s have the highest mortality ratio, which gradually decreases thereafter [8]. However, whether there is an actual decline in mortality is difficult to confirm since the studies that were compared have different inclusion criteria, including patients with different causes of hypopituitarism that have received dissimilar treatments. In fact, time trends of mortality in patients with pituitary diseases have not been studied previously in a single cohort.

The primary aim of this nationwide study was, therefore, to analyze whether mortality in patients with NFPA has changed during the last two decades. We also wanted to investigate whether mortality in men and women is affected by the length of the follow-up period (latency periods). Based on the above mentioned improvements in diagnosis and treatment of patients with pituitary disorders, we hypothesized that mortality has decreased over time.

### Subjects and methods

#### Study design

The Swedish National Patient Registry (Patient Registry) was used to identify patients with NFPA diagnosis in Sweden. The Patient Registry achieved national coverage in 1987 and contains information from every patient visit within the Swedish hospital system. The causes of death were retrieved from the Swedish National Cause of Death Registry (Cause of Death Registry), which reached national coverage in 1952. By using the unique Swedish personal identification number, an individual patient can be followed throughout life in the Swedish National Health registries. The National Board of Health and Welfare secures high quality for the Patient Registry and Cause of Death Registry [15, 16]. To identify patients with NFPA diagnosis we used a combination of identification criteria to ensure a high quality in the selection of the patients. The identification process has been previously described in detail in two studies when mortality and morbidity in the Swedish national cohort of patients with NFPA was studied [5, 9]. Briefly, patients included in the study should either have been diagnosed with NFPA [*International Classification of Diseases*, 10th revision (ICD-10) code D35.2)] at a medical/endocrine care unit or at a neurological or neurosurgical care unit. This method of combined criteria for identification (both diagnostic codes and department codes) has previously been shown to result in a high sensitivity and specificity when selecting patients with pituitary diseases in epidemiological research in the Nordic countries [17, 18]. For the studied cohort of patients, the internal validation of the identification process resulted in a positive predictive value of 91 % for the NFPA diagnosis [5, 9]. Other non-functioning pituitary tumors, mostly cystic pituitary tumors, were found in 4 % of the patients. Only 1 % of the patients had a lesion not affecting the sella region. The validation process has previously been described in detail [5, 9].

Patients with NFPA were identified in the Patient Registry from January 1, 1997 (when ICD-10 classification was implemented in Sweden) to December 31, 2011. The date of the NFPA diagnosis was also gathered from the Patient Registry, between January 1, 1987 to December 31, 2011 (including both ICD-9 and ICD-10 classification). All patients that were first diagnosed with an NFPA before January 1, 1997 were excluded from the cohort. The time at risk for the patients with NFPA started on the date of diagnosis (date of the first registered NFPA diagnosis). The time at risk, in which patients were studied regarding mortality, ended on the date of death or at the end of the study (December 31, 2014). Date of birth, gender, diagnoses of hypopituitarism, and diabetes insipidus (DI) as well as pituitary tumor treatments were collected from the Patient Registry. The degree of hypopituitarism and its management were not available in the registry. Patients were divided into three groups depending on the time-period of their first diagnosis of NFPA (1997–2001, 2002–2006, and 2007–2011). In the analyses of mortality ratio for patients with tumor treatment, the time of risk started on the date of the tumor treatment. Data on mortality in the general population was gathered and analyzed in the same manner as for the patients.

### Ethics

The study was approved by the Regional Ethical Review Board in Gothenburg, Sweden, and by the National Board of Health and Welfare, Sweden.

### Statistical analysis

Person-years at risk were calculated from study inclusion to death or end of study and stratified according to gender, 5 year age groups, and 1 year calendar periods. The expected number of cases for each stratum was calculated using the general Swedish population as reference. The observed number of deaths among subjects with NFPA was compared to those expected in the general population using standardized mortality ratios (SMRs). Ninety-five percent confidence intervals (CIs) were calculated assuming a Poisson distribution of the observed numbers. Subgroup analyses for different time-periods of diagnosis, gender, and treatment were performed. SMRs for non-overlapping subgroups were compared to each other [19]. To analyze possible trends and differences in between groups regarding diagnosis of hypopituitarism and DI as well as treatment patterns, Chi-square analyses were used with Linear-by-Linear Association tests and Pearson tests, respectively. Finally, the effect of different length of follow-up (different latency periods: 0–5, >5–10, and >10–18 years), i.e. the time between date of diagnosis and time of death, on mortality was analyzed [20]. IBM SPSS (version 21; SPSS Institute) and STATA/SE (version 12.1; StataCorp) software were used for the statistical analyses.

## Results

Data from 2473 patients with NFPA were included in the analysis: 1310 men and 1163 women. The mean (±SD) age at diagnosis was 58.9 ± 16.8 years. The mean (range) follow-up time was 8.3 (0–18) years, resulting in a total patient-years at risk of 20,517 (Table 1). A total of 1303 (53 %) patients had been treated surgically, including 771 (59 %) men and 532 (46 %) women (*P* < 0.001). One-hundred-and-fifteen patients (5 %) had received pituitary radiotherapy with no significant difference between men (n = 61; 5 %) and women (n = 54; 5 %). Of 1263 patients who were diagnosed with hypopituitarism, 795 were men and 468 women (61 and 40 % of men and women respectively; *P* < 0.001).

**Table 1:** Characteristics of patients with non-functioning pituitary adenoma

Characteristics	Total (N = 2473)
Gender, n (%)	
Men	1310 (53)
Women	1163 (47)
Mean age at diagnosis, years (range)	58.9 (1–97)
Men, mean ± SD	61.0 ± 15.7
Women, mean ± SD	56.6 ± 17.7
Diagnosed with hypopituitarism, n (%)	1263 (51)
Diagnosed with diabetes insipidus, n (%)	122 (4.9)
Mean patient-years at risk per patient, year (range)	8.3 (0–18)
Patient-years at risk in the study	20,517
Time at diagnosis	
1997–2001	607 (25)
2002–2006	881 (36)
2007–2011	985 (40)
Treated with surgery, n (%)	1303 (53)
Treated with radiotherapy, n (%)	115 (5)

Six-hundred-and-seven patients (25 %) were diagnosed between 1997 and 2001, 881 (36 %) between 2002 and 2006, and 985 (40 %) between 2007 and 2011. The prevalence of hypopituitarism declined during the three time periods in both men and women (Table 2). The prevalence of diabetes insipidus and the use of pituitary surgery and radiotherapy remained stable during the study period in both genders.

**Table 2:** Hypopituitarism, diabetes insipidus, and treatment with pituitary surgery and radiotherapy in patients with non-functioning pituitary adenoma in Sweden followed between 1997 and 2014

Outcome	No. of patients (%)			
	Diagnosed between 1997–2001 (n = 607)	Diagnosed between 2002–2006 (n = 881)	Diagnosed between 2007–2011 (n = 985)	Trend analysis, *P* value
Radiotherapy				
Total	28 (4.6)	52 (5.9)	35 (3.6)	0.20
Men	12 (3.8)	31 (6.8)	18 (3.4)	0.51
Women	16 (5.5)	21 (5.0)	17 (3.8)	0.29
Surgery				
Total	313 (52)	479 (54)	511 (52)	0.96
Men	176 (56)	281 (61)	314 (59)	0.55
Women	137 (47)	198 (47)	197 (44)	0.37
Hypopituitarism				
Total	390 (64)	466 (53)	407 (41)	<0.0001
Men	242 (77)	289 (63)	264 (49)	<0.0001
Women	148 (51)	177 (42)	143 (32)	<0.0001
Diabetes insipidus				
Total	31 (5.1)	50 (5.7)	41 (4.2)	0.32
Men	15 (4.7)	17 (3.7)	16 (3.0)	0.19
Women	16 (5.5)	33 (7.8)	25 (5.6)	0.88

**Fig. 1:**
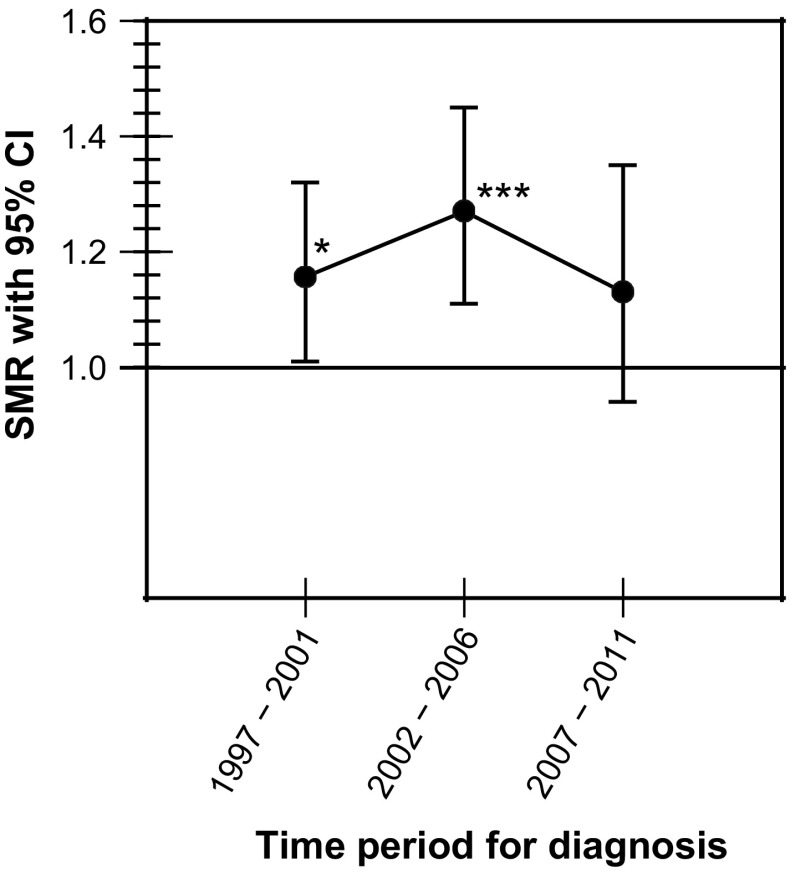
Overall mortality in patients with non-functioning pituitary adenoma in Sweden followed between 1997 and 2014. *P < 0.05; ***P < 0.001

**Fig. 2:**
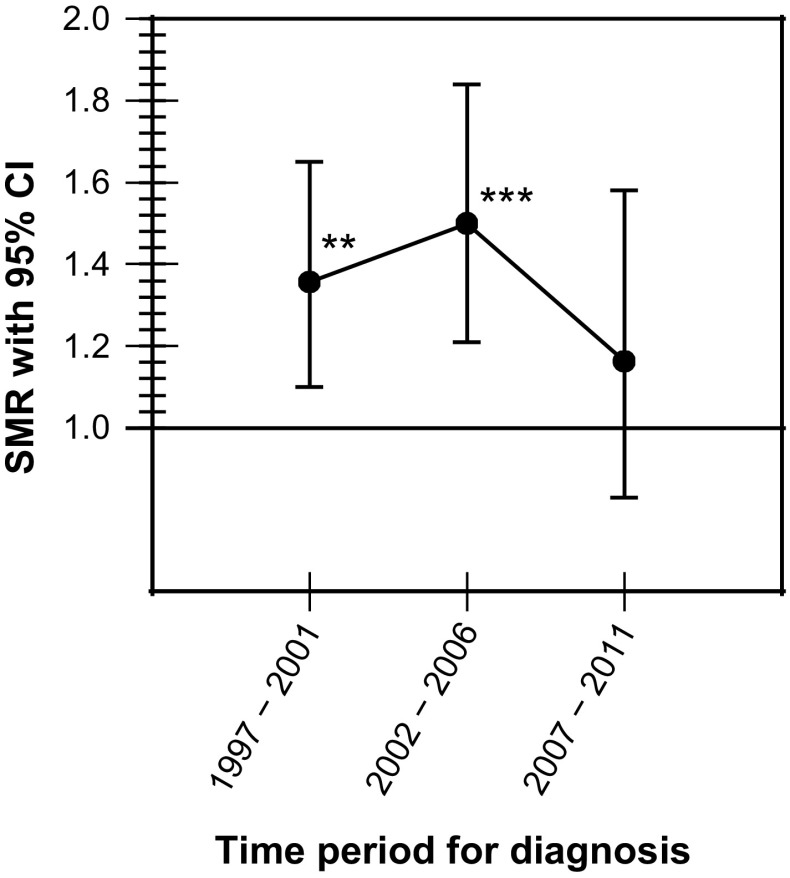
Overall mortality in women with non-functioning pituitary adenoma in Sweden followed between 1997 and 2014. **P < 0.01; ***P < 0.001

The overall mortality ratio was increased in women [SMR 1.37 (95 % CI 1.20–1.56)] but not in men [SMR 1.09 (95 % CI 0.97–1.22)] (Table 3). The overall mortality ratio was increased in patients diagnosed between 1997 and 2001 as well as between 2002 and 2006 but seemed to return to normal in those diagnosed between 2007 and 2011 (Fig. 1). In women, the mortality ratio was increased during the first two time periods but not in those diagnosed between 2007 and 2011 (Fig. 2). The mortality ratio in men remained stable throughout all three periods of diagnosis and did not differ from the general population (Table 3). During the last time period, 2007–2011, the mortality ratio did not differ between men and women.

**Table 3:** Time trends of mortality in patients with non-functioning pituitary adenoma in Sweden followed between 1997 and 2014

Outcome	Expected no. of deaths	Observed no. of deaths	Standardized mortality ratio (95 % CI)	*P* value
*Overall mortality*				
Men^a^	293.4	320	1.09 (0.97–1.22)	0.13
Women^a^	168.1	230	1.37 (1.20–1.55)	<0.0001
*Time trends depending on year of diagnosis*				
Overall				
1997–2001	187.8	217	1.16 (1.01–1.32)	0.040
2002–2006	167.7	213	1.27 (1.11–1.45)	0.0009
2007–2011	106.0	120	1.13 (0.94–1.35)	0.19
Men				
1997–2001	114.1	117	1.03 (0.85–1.23)	0.81
2002–2006	107.7	123	1.14 (0.95–1.36)	0.16
2007–2011	71.6	80	1.12 (0.89–1.39)	0.35
Women				
1997–2001	73.7	100	1.36 (1.10–1.65)	0.004
2002–2006	60.0	90	1.50 (1.21–1.84)	0.0004
2007–2011	34.4	40	1.16 (0.83–1.58)	0.38
Patients treated with surgery				
1997–2001	86.5	100	1.16 (0.94–1.41)	0.17
2002–2006	85.1	102	1.20 (0.98–1.45)	0.082
2007–2011	42.9	42	0.98 (0.71–1.32)	0.97
Patients treated with radiotherapy				
1997–2001	3.1	13	4.19 (2.23–7.16)	<0.0001
2002–2006	5.8	16	2.76 (1.58–4.48)	0.007
2007–2011	2.5	13	5.22 (2.78–8.93)	<0.0001

^a^ Standardized mortality ratio significantly (*P* = 0.011) different between men and women

**Fig. 3:**
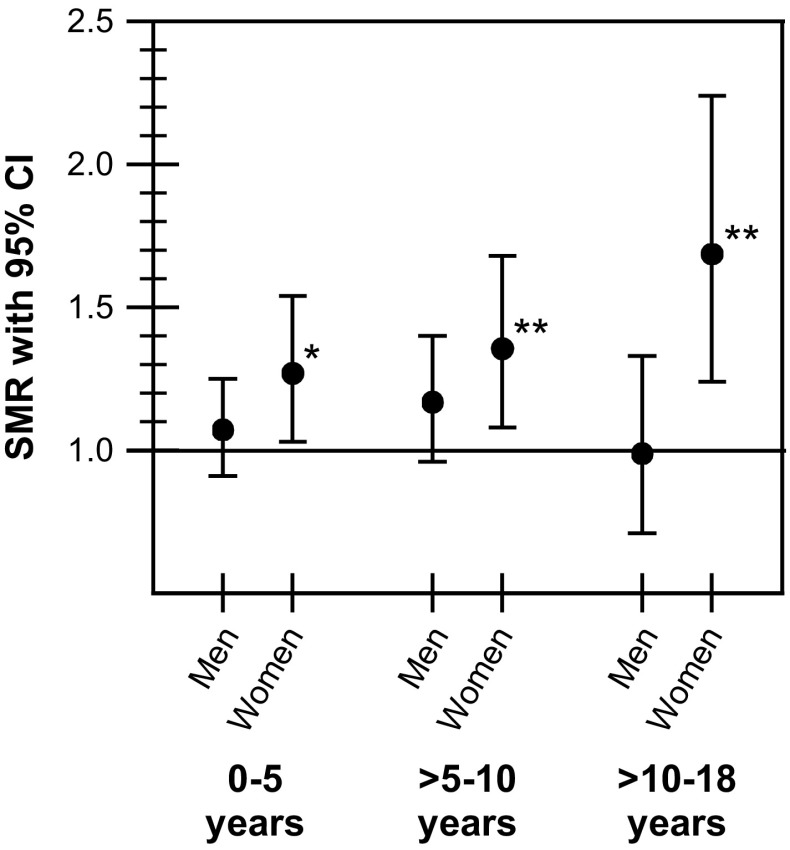
Mortality in men and women with non-functioning pituitary adenoma analyzed depending on latency periods (0–5, >5–10, and >10–18 years). *P < 0.05; **P < 0.01

Mortality in men with NFPA in relation to duration of follow-up time (latency periods) did not differ from the mortality in the background population (Fig. 3). In women, in contrast, the mortality was increased during all three follow-up periods, with SMR being the lowest for the shortest latency period 0–5 years [1.27 (95 % CI 1.03–1.54)] and the highest for the longest latency period >10–18 years [1.69 (95 % CI 1.24–2.24)]. The mortality during the longest latency period (>10–18 years) was significantly higher for woman compared to men (*P* = 0.015).

## Discussion

In this study we present the first analysis of time trends of mortality in patients with NFPA. The overall mortality rate in women was higher than in the general population as well as compared to men with NFPA. Interestingly, and in accordance with our hypothesis, the mortality has decreased significantly in women who were diagnosed with NFPA during the most recent time period. In fact, the mortality in women during the last time period of diagnosis (2007–2011) was similar with that in men with NFPA, as well as not different compared to the mortality in the general population.

There may be several explanations for our results showing declining mortality in women with NFPA. As we and others have previously shown, patients with pituitary hypopituitarism, especially women, have increased mortality [1, 3, 5, 8]. We have recently also demonstrated increased incidence of myocardial and cerebral infarction in women with hypopituitarism due to NFPA as compared to women without hormonal deficiencies [9]. In the current study a declining prevalence of hypopituitarism was observed. Half of the women diagnosed with NFPA during the first time period had hypopituitarism, 42 % during the second period, and one-third during the last period. Thus, a plausible explanation for the declining mortality may be the lower prevalence of pituitary insufficiency due to the development of more accurate and less traumatic surgical techniques.

The repetitive finding that mortality in women with pituitary disorders is higher than in men is worrisome. Since hypopituitarism was more prevalent in men than in women, one might speculate whether women are more rarely tested, and subsequently treated, for growth hormone deficiency and hypogonadism. Also, the clear difference in the frequency of pituitary surgery between the genders may indicate that the women receive a less directed, and inappropriate, tumor treatment. It is well known that women with coronary heart disease are diagnosed later, undergo more seldom coronary artery surgery, and have worse outcome than men [21, 22]. Similar gender differences have been observed concerning diagnosis, morbidity, and mortality in patients with diabetes mellitus [23]. It is therefore probable that inferior treatment and surveillance, may at least contribute, to the worse outcome in women with NFPA.

An interesting finding in the current study was the gradually increasing mortality in women in relation to increasing duration of the follow-up time. A likely explanation is the protracted and cumulative effect of underuse and/or inadequate hormone replacement therapy in women with hypopituitarism. This finding also emphasizes the importance of a sufficiently long follow-up time in epidemiological research in general.

The prevalence of pituitary adenomas diagnosed *post mortem* is around 10 % [24]. In unselected populations the prevalence of pituitary incidentaloma, i.e. adenomas diagnosed accidentally on imaging, range between 10 and 38 % [25]. In the current study, improved imaging techniques during the last two decades may have resulted in an increased incidence of small pituitary incidentalomas, i.e. tumors that have a minimal influence on health in general. The unchanged frequency of pituitary surgery and radiotherapy during the study, however, argues against this possible explanation. Nevertheless, the lack of information on tumor size in our cohort is a limitation that must be taken into account as a possible confounder.

The major strength of this study is the large number of patients, in fact the largest cohort of NFPA patients studied to date, and the long follow-up period. Additionally, after a validation of the diagnosis more than 90 % had a proven NFPA, which is a high proportion in an epidemiological study [5]. This study has, however, some limitations. Since this is a register-based study, detailed information on the diagnosis and treatment of pituitary insufficiency was not available. Analysis of time trends concerning prevalence and treatment of growth hormone deficiency, adrenal insufficiency, hypothyroidism, and hypogonadism were therefore not possible. Similarly, detailed information on pituitary surgery techniques was not available. Also, a potential bias in the latency period analysis could be that only patients diagnosed in the earliest period of the study (1997–2001) have an extensive duration of follow-up. Nevertheless, male patients do not display the same trend of increased mortality as women do, which argues against the influence of this potential bias.

In conclusion, while mortality in men with NFPA remains normal and stable during the last two decades, mortality in women has declined. Decreasing prevalence of pituitary insufficiency may be a plausible explanation for this positive development.
